# Increased Abundance of Plasmacytoid Dendritic Cells and Interferon-Alpha Induces Plasma Cell Differentiation in Patients of IgA Nephropathy

**DOI:** 10.1155/2017/4532409

**Published:** 2017-12-18

**Authors:** Nuoyan Zheng, Bing Wang, Jinjin Fan, Ning Luo, Qingyu Kong, Hongjian Ye, Jiqin Zhang, Hongyan Ming, Xueqing Yu

**Affiliations:** ^1^Translational Medical Center, The First Affiliated Hospital of Sun Yat-sen University, Guangzhou, China; ^2^Department of Nephrology, The First Affiliated Hospital of Sun Yat-sen University, Guangzhou, China; ^3^International Travel Health Care Center, Entry-Exit Inspection and Quarantine Bureau, Guangzhou, Guangdong Province, China

## Abstract

The roles of pDC and IFN-*α* have not been well defined in IgA nephropathy (IgAN). In this study, we investigated the abundance of pDCs and IFN-*α* in IgAN patients and the response of peripheral blood mononuclear cells (PBMCs) after stimulation of the pDC-preferred TLR9 ligand CpG2216. The effects of IFN-*α* on plasma cell differentiation and leukocyte migration were also investigated. Here, we found that the percentages of pDCs were increased in PBMCs of IgAN patients, than in those of healthy controls. Plasma levels of IFN-*α* proteins and abundance of plasma cells were higher in IgAN patients than in healthy donors. Plasma IFN-*α* levels were positively associated with proteinuria, renal IgM deposition, and renal tubular atrophy/interstitial fibrosis grade in IgAN patients. Ex vivo activation of TLR9 on pDCs resulted in increased IFN-*α* production and enhanced plasma cell differentiation in IgAN patients as compared with healthy donors. IFN-*α* treatment led to increased plasma cell differentiation *in vitro*. IFN-*α* also significantly promoted expression of chemokines IP-10 and MCP-1 in human mesangial cells, which subsequently facilitated the transendothelial migration of human CD4^+^ and CD14^+^ cells. In conclusion, pDC and its secreted cytokine IFN-*α* may play important roles in pathological changes of IgA nephropathy.

## 1. Introduction

IgA nephropathy (IgAN) was the most common form of primary glomerulonephritis worldwide, and it is well accepted that the interaction between genetic, epigenetic, and environmental factors may synergistically contribute to the pathogenesis of IgAN [1–4]. One of the environmental-related factors, infection of pathogens, has been well acknowledged for the pathogenesis of IgAN; about 30% of IgAN patients suffered from disease onset and/or progression after upper respiratory infection or gastrointestinal infection [5, 6]. Bacterial and viral antigens (such as *Staphylococcus aureus*, *Haemophilus parainfluenzae*, *Epstein-Barr* virus, and retroviruses) have been detected in the renal specimens of IgAN patients [6–8]. In defending against these foreign pathogens, toll-like receptor (TLRs) family members are critical by triggering strong innate and adaptive immunity in the host [9]. Upon recognition of pathogen-associated molecular patterns, TLRs recruit adaptor molecules such as MyD88 and TRIF to initiate downstream signaling events which leads to the secretion of inflammatory cytokines, type I IFN, chemokines, and so forth and subsequently causes a serial of inflammatory response including recruitment of neutrophils, activation of macrophages, maturation of dendritic cells, and induced expression of serials of IFN-stimulated genes for killing infected pathogens [10, 11]. Although TLRs are very effective for fighting pathogens, they may also act as a double-blade sword since enduring activation of TLRs may cause uncontrolled inflammatory responses and tissue damages [12–14]. In addition to recognizing exogenous ligands, TLRs may also recognize endogenous ligands including self-proteins and endogenous nucleic acids then elicit autoimmune disease as in rheumatoid arthritis and systemic lupus erythematosus [15]. The important roles of TLR4 and TLR9 in IgAN have been studied by several groups, pointing out that their expression was associated with disease severity and even the pathogenesis of IgAN [16–20], but the underlying mechanism of how TLRs led to the pathogenesis of IgA nephropathy is not fully elucidated.

Plasmacytoid dendritic cells (pDCs) are relatively new in the category of immune cell types, which were first fully characterized in 1999. Given the fact that pDCs are very rare, accounting for 0.3–0.5% of the human peripheral blood cells, the biology and function of pDCs in diseases are incompletely understood [21]. The immunophenotype markers of pDCs in the blood have been reported in several studies and characterized as Lin(CD3/14/56/19/20/16)^−^CD123^high^CD11c^−^HLA-DR^+^ [22–25]. In addition, specific markers BDCA-2 and BDCA-4 are also exclusively expressed on pDCs which reside in blood and bone marrow [26]. The primary and unique function of pDCs is the secretion of type I interferon (IFN-*α*/*β*) in response to virus and/or virus-derived nucleic acids and/or apoptotic cell-derived nucleic acids [27–29]. Studies revealed that TLRs expression pattern in pDCs is restricted to TLR7 and TLR9 [30] and their activation leads to substantial production of IFN-*α* [21]. The endosome-located TLR7 and TLR9 are, respectively, responsible for sensing single-stranded RNA and unmethylated 5′-cytosine-phosphate-guanine-3′ (CpG) DNA motif, later eliciting the production of large amount of IFN-*α* in pDCs which can be up to 1000-fold of other cell types [31]. In fact, IFN-*α* production in response to bacteria CpG motif challenge *in vivo* is exclusively mediated by pDCs, which pass through the TLR9/MyD88/IRF7 signaling pathway [32, 33]. There are three types of CpG DNA (CpG-A, CpG-B, and CpG-C) targeting different cell populations and result in different downstream events. Type CpG-A is very potent in provoking type I interferon production in pDCs but very weak in stimulating B cell response, while it enhances the expression of IRF7 and provides a positive feedback loop for IFN-*α* production via IFNAR [34–36]. CpG-B is superior in terms of B cell stimulation, whereas CpG-C combines the B cell-activating properties of CpG-B and the IFN-*α*-inducing properties of CpG-A [36, 37].

Here, we investigated the possible roles of pDCs in IgAN by studying its abundance, secretion of IFN-*α*, and the capacity of stimulating B cell activation and differentiation. We found that pDCs and plasma cells were significantly more abundant in PBMCs of IgAN patients than in those of healthy controls; meanwhile, plasma IFN-*α* levels were about 16-fold higher in IgAN patients than in healthy controls. When activated upon pDC-preferred TLR9 ligand CpG2216 (type CpG-A) ex vivo, PBMCs from IgAN patients secreted more IFN-*α* than those from control groups, along with augmented plasma cell differentiation. Consistently, IFN-*α* can stimulate the differentiation of plasma cells, but not the proliferation of B cells *in vitro*. Furthermore, we found that IFN-*α* promoted the production of chemokines MCP-1 and IP-10 from human mesangial cells, which resulted in increased transendothelial migration of human CD4^+^ and CD14^+^ cells. This study reveals new pathological roles of pDCs and IFN-*α* in IgAN, indicating new therapeutic targets for this complex disease.

## 2. Material and Methods

### 2.1. Study Groups

Biopsy proven, primary IgAN patients were enrolled in The First Affiliated Hospital of Sun Yat-sen University for this study. Healthy donors and patients with lupus nephritis were enrolled as control groups. All recruited donors were of Chinese Han population. All healthy participants are negative for hematuria and proteinuria, with normal renal and liver function, without a past history of kidney diseases. There were no symptoms of infection observed among all participants 4 days before and 3 days after the blood sample collection. None of the patients were treated with steroids and/or immunosuppressive drugs within one year. Patients diagnosed with end-stage renal disease were excluded in this study. All participants were in conformity with written informed consent. This study obtained approval from the ethics review committee of The First Affiliated Hospital of Sun Yat-sen University, Guangzhou, China. This study was conducted in accordance with the guidelines proposed in the Declaration of Helsinki. All adults gave their written informed consent.

The renal histopathology of IgAN patients was classified according to the Oxford classifications [38]. Renal histopathology from all patients was scored by 2 renal pathologists blinded to the clinical data for the 4 pathological variables: the mesangial hypercellularity (M0 ≤ 0.5 or M1 ≥ 0.5), the segmental glomerulosclerosis (S0 absent or S1 present), the endocapillary hypercellularity (E0 absent or E1 present), and the tubular atrophy/interstitial fibrosis (T0 ≤ 25%; T1 26–50%; T2 > 50%) [39, 40].

### 2.2. Reagents

A reverse transcription kit (TAKARA, DRR037A) and real-time master kit (DRR014A) were purchased from Takara Bio Inc. (Otsu, Japan); Trizol reagent (15596018) and DNaseI (AM2235) were from Thermo Fisher Scientific Inc. (Waltham, MA, USA). Antibodies against CD markers (CD3, CD4, CD14, CD19, CD16, CD56, CD20, CD123, HLA-DR, CD11c, and CD38) were purchased from the eBioscience company (San Diego, CA, USA). An IFN-*α* ELISA kit (3425-1H-6) was provided by the Mabtech company (Cincinnati, Ohio, USA), and recombinant human IFN-*α* and TNF-*α* were purchased from PeproTech Inc. (Westlake Village, CA, USA). CpG2216 oligonucleotides were synthesized by Thermo Fisher Scientific Inc. (Waltham, MA, USA). Ig subset antibodies for IgA1, IgA2, and IgG ELISA were purchased from Abcam Inc. (Cambridge, UK), LifeSpan BioSciences Inc. (Seattle, WA, USA), and DAKO Inc. (Santa Clara, CA, USA). Anti-IFN-*α* and anti-TLR9 antibody for immunofluorescence staining were purchased from Thermo Fisher Scientific Inc. (Waltham, MA, USA) and Santa Cruz Biotechnology Inc. (Santa Cruz, CA, USA), respectively.

### 2.3. Analysis of Plasma Cells and pDCs in PBMCs by Flow Cytometry

PBMCs from venous blood with anticoagulant EDTA-K2 were enriched by Ficoll-paque density centrifugation (800 ×g for 20 minutes). Cells were then washed for four times with phosphate-buffered saline (PBS) with 2 mM EDTA. For pDC analysis, human PBMCs were stained in a combination of fluorochrome-conjugated antibodies for Lin markers (CD3-FITC, CD14-FITC, CD19-FITC, CD20-FITC, CD16-FITC, and CD56-FITC), CD11c-APC, CD123-PE, and HLA-DR-PE Cy7 at 4°C for 30 minutes. After washing with PBS/3% FBS/2 mM EDTA, the cells were analyzed with flow cytometry using a Moflo instrument (Beckman Coulter, Brea, CA, USA). The population of pDC was characterized as surface labeling of Lin^−^CD123^high^HLADR^+^CD11c^−^. For plasma cell analysis in fresh PBMCs, the cells were collected and stained in a combination of fluorochrome-conjugated antibodies (CD19-PE, CD20-FITC, and CD38-PE Cy7) at 4°C for 30 minutes and then analyzed with flow cytometry.

### 2.4. IFN-*α* and IgA1 Measurement with Enzyme-Linked Immunosorbent Assay (ELISA)

The levels of IFN-*α* in plasma samples and cell culture supernatant were measured by ELISA kits according to the manufacturer's instructions with a detection range of 2–1000 pg/ml. For Ig measurement, samples or Ig subset standards were added to 96-well microplates coated with capture antibody; subsequently, secondary anti-human Ig antibody was added as detection antibody. The plates were incubated at 37°C for 3 h, followed by peroxidase-conjugated streptavidin at 37°C for 60 min after washing with PBS. The color was developed with 3,3′,5,5′-tetramethylbenzidine (TMB), and the reaction was stopped by adding 100 *μ*l of 2 N HCl for 5 min after adding TMB. Optical densities were measured by a microplate reader (Spectra Max M5, Molecular Devices) at 450 nm wavelength. Results were analyzed statistically together with clinical parameters and renal histopathology to explore the possible relationship between IFN-*α* and disease severity.

### 2.5. Immunofluorescence Staining of Tonsil Samples from IgAN Patients

IgAN patients who suffered from tonsillitis and received tonsillectomy were recruited for this study. 4% paraffin-embedded sections of tonsil samples (4 *μ*m thickness) were deparaffinized with xylene and rehydrated through a graded ethanol series. Before staining, slides were preincubated 1 h with PBS/0.3% Triton X-100/3% bovine serum albumin. Sections were stained with anti-IFN-*α* and anti-TLR9 antibody for 4 h. Fluorescence signal was amplified with fluorochrome-conjugated secondary antibody, followed by a subsequent DAPI staining and extensive washing with PBS. Sections were observed with a Zeiss LSM 510 Meta Duo Confocal microscope.

### 2.6. Ex Vivo Stimulation of PBMCs with CpG2216 Oligonucleotides

PBMCs isolated from IgAN patients and healthy donors were separated with ficoll gradient centrifugation and resuspended in RPMI 1640 complete culture medium as described [41]. Cells were plated in 96-well culture plates at the density of 2.5 × 10^5^/well and cultured at 37°C in a 5% CO_2_-humidified incubator. Cells were stimulated with 5 *μ*g/ml CpG2216 (5′GGgggacgatcgtcgGGGGG3′, the upper case letters indicating phosphorothioate backbones). The supernatant of cells was harvested 24 h after CpG2216 stimulation and subjected to ELISA for IFN-*α* measurement. The supernatant was harvested at day 13 after stimulation for Ig analysis with ELISA. The cells were collected at day 7 after CpG2216 stimulation and stained with fluorochrome-conjugated antibodies (anti-CD19-APC, anti-CD20-APC-Cy7, and anti-CD38-PE Cy7) for plasma cell analysis with flow cytometry.

### 2.7. The Effect of IFN-*α* on Plasma Cell Differentiation and IgA1 Synthesis

PBMCs isolated from healthy donors were cultured in RPMI 1640 complete culture medium with or without recombinant IFN-*α* treatment (2000 IU/ml). Six days after IFN-*α* stimulation, cells were collected and analyzed for plasma cell differentiation as described above; meanwhile, culture supernatant was collected for IgA1 measurement.

### 2.8. The Effects of IFN-*α* on Gene Expression of Human Mesangial Cells

The immortalized human mesangial cell line (HMC) was kindly provided by F. X. Huang (Sun Yat-Sen University, Guangzhou, China) [42] and grown in RPMI 1640 supplemented with 10% (*v*/*v*) fetal bovine serum at 37°C in a 5% CO_2_-humidified incubator. Equal numbers of mesangial cells were cultured until 80–90% confluence and subjected to serum starvation for 4–6 h before treatment. Recombinant IFN-*α* (4000 IU/ml) was added to the cell culture medium and incubated with cells for different time periods. Cells were then collected for RNA extraction and later for gene expression analysis.

Samples of total RNA of cells were extracted using Trizol reagent following the manufacturer's instructions. Samples of cDNA were synthesized from RNA using a reverse transcriptase kit according to the manufacturer's manual. Real-time PCR was performed to measure gene expression levels of TGF-*β*, VEGFA, RANTES, ICAM-1, IL-6, IFNAR1, IP-10, iNOS, TNF-*α*, IL-1*β*, PDGF-BB, MCP-1, MCP3, VCAM-1, MIP-1b, and CCL17 in an ABI 7900HT instrument (Applied Biosystems, South San Francisco, CA, USA) for 40 cycles (95°C for 30 sec, 58°C for 30 sec, and 72°C for 30 sec for each cycle). Data were expressed as fold changes using the ΔΔCt method [43] with GAPDH as an internal control.

### 2.9. Transendothelial Migration (TEM) of CD14^+^ and CD4^+^ Cells Mediated by Conditioned Medium from HMC Treated with IFN-*α*

#### 2.9.1. Preparation of CD14^+^ and CD4^+^ Cells

PBMCs obtained from healthy donors were separated with ficoll gradient centrifugation as described above. CD14^+^ and CD4^+^ cells were enriched from harvested PBMCs by sorting in Moflo instrument after cells were labeled with fluorochrome-conjugated antibodies against a CD14 or CD4 marker. CD14^+^ monocytes/macrophages were resuspended at a density of 1 × 10^6^ cells/ml in RPMI 1640/0.5% FBS medium. CD4^+^ T cells were stimulated with anti-CD3 antibody (5 *μ*g/ml) overnight and resuspended at 1 × 10^7^ cells/ml in RPMI 1640/0.5%FBS medium.

#### 2.9.2. Preparation of Conditioned Medium from Human Mesangial Cells Treated with IFN-*α*

Human mesangial cells in RPMI 1640/10% FBS were treated with IFN-*α* at 4000 IU/ml for 6 h. Cells were washed once with plain culture medium, and culture was resumed in fresh RPMI 1640 with 0.5% fetal bovine serum for 16–18 h. Conditioned medium was collected by centrifugation at 1600 rpm for 6 min at 4°C. Aliquots were stored at −20°C for future use.

Human umbilical vascular endothelial cells (HUVECs) were prepared by collagenase treatment of umbilical cords as previously described [44]. Primary endothelial cells at passage three were plated at a density of 2 × 10^4^ cells/well onto gelatin-coated transwells (5 *μ*M pore size, cat. 3421, Corning, New York, USA) and cultured until 100% confluence in ECM medium with 20% FBS, followed by an overnight stimulation of 10 ng/ml TNF-*α*. HUVECs were washed once and then balanced for at least 1 hr with RPMI 1640/0.5% FBS at 37°C. Conditioned medium harvested from HMC was added to the bottom chambers of a transwell (0.6 ml/well). CD14^+^ and CD4^+^ cells were, respectively, added to the top wells at the density of 1 × 10^5^ cells/100 *μ*l and 1 × 10^6^ cells/100 *μ*l. A transwell plate was incubated in a CO_2_ incubator at 37°C, allowing cell migration for 6–12 h. Cells which migrated into the bottom wells were harvested with trypsin and centrifugation, followed by Hoechst staining. Migrated CD14^+^ or CD4^+^ cells were examined with a fluorescence microscopy (Leica).

### 2.10. Statistical Analysis

Data was expressed as mean ± standard error value and treated with Student's *t*-test. For data of three and more groups were involved, we carried out one-way ANOVA and further post hoc test when *p* < 0.05. The correlation was tested with Pearson's correlation coefficients and presented with a scatter plot. Statistical analysis was performed with the SPSS 18.0 software. All statistical assessments were two-sided using a significance value of less than 0.05.

## 3. Results

### 3.1. Plasmacytoid Dendritic Cells and Its Signature Cytokine IFN-*α* Were More Abundant in Peripheral Blood of IgAN Patients

**Figure 1:**
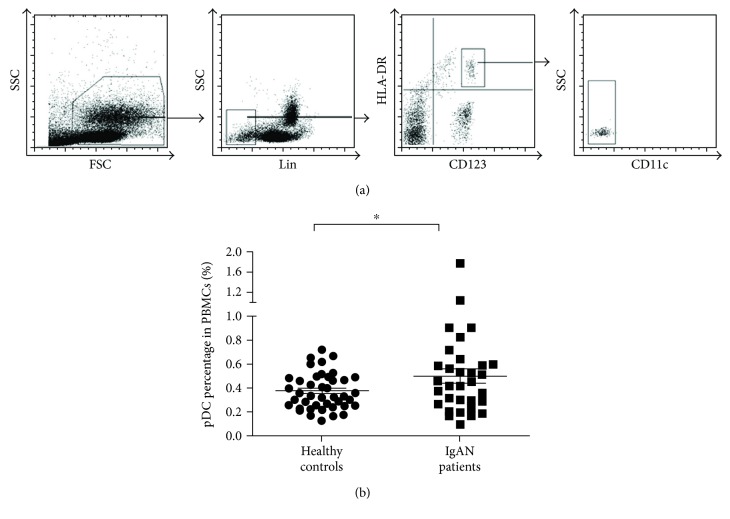
The percentages of pDCs were increased in PBMCs of IgAN patients. Peripheral blood mononuclear cells (PBMCs) of donors were prepared by ficoll-paque density centrifugation and subjected to flow cytometry analysis. (a) The surface phenotype of pDCs was defined as Lin^−^CD123^high^HLA-DR^+^CD11c^−^. (b) The percentages of pDCs in PBMCs from IgAN patients and healthy donors were analyzed. ^∗^ *p* < 0.05.

**Figure 2:**
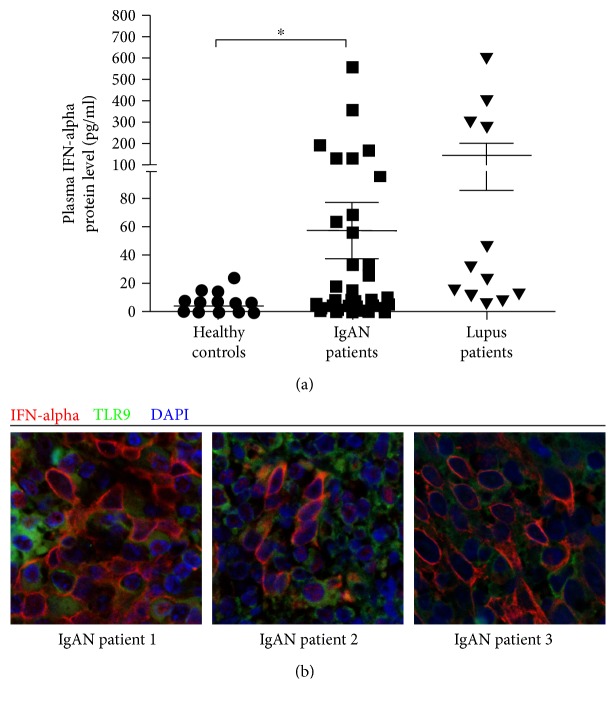
(a) Concentrations of IFN-*α* proteins in plasma were measured in different groups. PBMCs of different donors were prepared by ficoll-paque density centrifugation, and plasma in upper layer was harvested for ELISA analysis. ^∗^ *p* < 0.05. (b) Paraffin-embedded sections of tonsil from IgAN patients with tonsillitis were stained for IFN-*α* and TLR9 expression.

As we investigated the abundance of pDCs in PBMCs of IgAN patients and healthy controls, multiple CD markers labeling (Lin(CD3/14/19/20/16/56)^−^CD123^high^HLA-DR^+^CD11c^−^) were used to characterize this cell population (Figure 1(a)). The percentage of pDCs in PBMCs was found significantly higher in IgAN patients than in healthy controls (0.51% versus 0.37%, *p* = 0.029) (Figure 1(b)). IFN-*α* is the most remarkable cytokine for pDC, and we found that plasma IFN-*α* levels were significantly upregulated in IgAN patients (56.9 ± 20.2 pg/ml) than in healthy donors (3.4 ± 1.0 pg/ml). Plasma levels of IFN-*α* in IgAN patients were lower than those in patients of lupus nephritis (143.7 ± 57.8 pg/ml) (Figure 2(a)). Furthermore, we examined the expression of IFN-*α* and TLR9 in tonsil samples of IgAN patients who suffered from tonsillitis. We found that IFN-*α* was evidently expressed in tonsillar cells of IgAN patients; meanwhile, TLR9 was richly expressed in IFN-*α*-positive cells (Figure 2(b)).

Demographic and clinical profile of IgAN patients and controls is listed in Table 1. When investigating the correlation among plasma IFN-*α* concentrations and clinical features, plasma IFN-*α* levels exhibited significant and positive association with 24-hour proteinuria (*r* = 0.34, *p* = 0.04) and anti-Dnase B titer (*r* = 0.48, *p* = 0.005) in patients with IgAN (Table 2). Regarding renal histopathology, we found that pDC percentages were positively associated with renal IgM deposition in IgAN patients (*r* = 0.45, *p* = 0.009). Also, plasma IFN-*α* concentrations were significantly associated with tubular atrophy/interstitial fibrosis grade (*r* = 0.37, *p* = 0.03), as well as renal IgM deposition (*r* = 0.34, *p* = 0.04) in IgAN patients (Table 2).

### 3.2. pDC-Preferred CpG Stimulation Induced IFN-*α* Secretion and Plasma Cell Differentiation in PBMCs of IgAN Patients

CpG2216 was a potent activator for TLR9 expressed on pDCs, and we employed it to study the response of PBMCs culture from IgAN patients. First, we confirm that IFN-*α* was mostly secreted by pDCs but not by other cells from PBMCs after CpG2216 stimulation (Supplemental Figure 1). Also, as shown in Supplemental Figure 2, CpG ODN 2216, but not control ODN, was able to provoke the synthesis of IFN-*α*; meanwhile, two reported inhibitory ODNs [45–47] of TLR9 suppressed CpG2216-mediated IFN-*α* secretion to less than 50% (Supplemental Figure 2).

**Figure 3:**
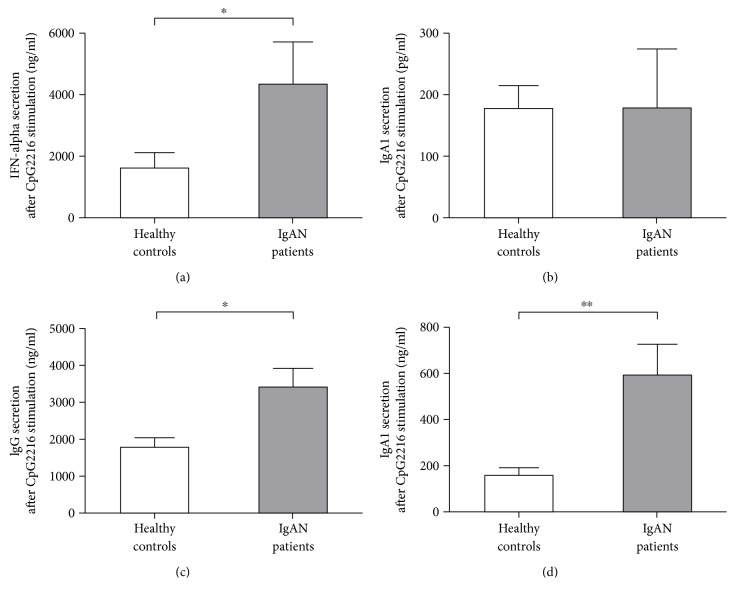
Secretion of IFN-*α* was increased in PBMCs of patients of IgAN after CpG2216 stimulation. PBMCs of different donors (healthy donors, *n* = 23; IgAN patients, *n* = 14) were prepared by ficoll-paque density centrifugation and cultured with pDC-preferred CpG2216 for different days, and the supernatant samples were collected for measuring IFN-*α*, IgA1, IgA2, and IgG levels. ^∗^ *p* < 0.05; ^∗∗^ *p* < 0.01.

When subjected to CpG2216 stimulation, PBMCs from IgAN patients secreted more IFN-*α* proteins (4338 ± 1374 pg/ml) into supernatant than those from healthy controls (1600 ± 508 pg/ml) (Figure 3(a)). Moreover, the secretion of IgG antibodies from PBMCs of IgAN patients was significantly stronger than that from healthy controls (3425 ± 525 versus 1788 ± 251 ng/ml) (Figure 3(c)). A similar result was found for IgA2 antibody secretion (593 ± 133 versus 155 ± 36 ng/ml) (Figure 3(d)), but not for IgA1 antibodies (Figure 3(b)).

**Figure 4:**
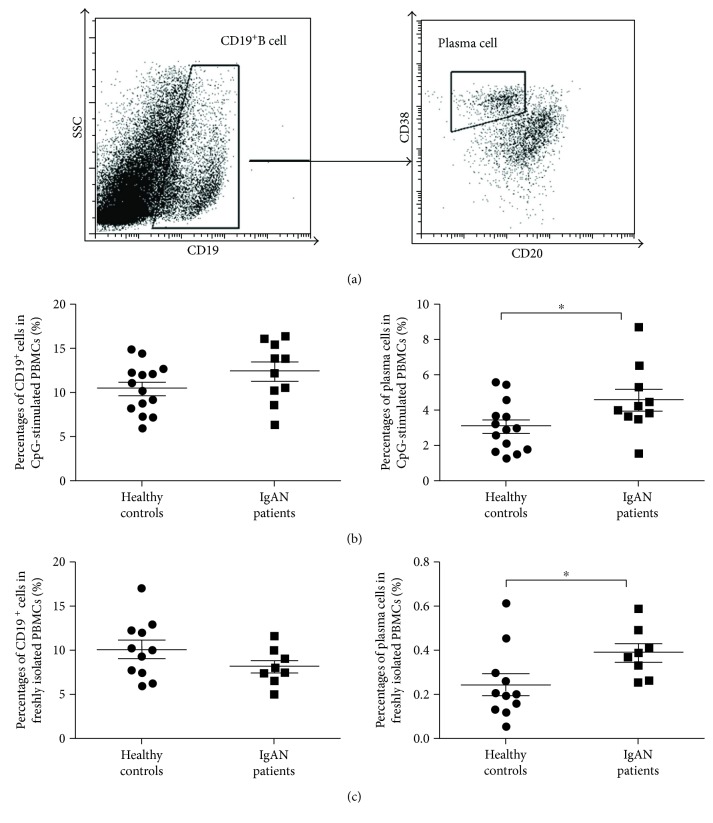
Plasma cell differentiation was augmented in CpG2216-activated PBMCs ex vivo. PBMCs of different donors were prepared by ficoll-paque density centrifugation and cultured with pDCs-preferred CpG2216 for 6 days, followed by surface marker labeling and analysis in flow cytometry. (a) CD19^+^ cells differentiated into plasma cells (CD19^+^CD38^high^CD20^low^) after CpG2216 stimulation for 6 days. (b) The percentage of CD19^+^ cells and plasma cells in ex vivo PBMCs culture after 6 days (healthy donors, *n* = 14; IgAN patients, *n* = 10). (c) The percentage of CD19^+^ cells and plasma cells in fresh PBMCs from IgAN patients and healthy controls (healthy donors, *n* = 11; IgAN patients, *n* = 8). ^∗^ *p* < 0.05.

We further investigated the regulation of plasma cell differentiation by TLR9 activation in PBMCs culture of IgAN patients. After CpG2216 stimulation for 6 days, CD19^+^ B cells became activated in PBMCs and differentiated into plasma cells by labeling as CD19^+^CD20^low^CD38^high^ (Figure 4(a)). In contrast to the CpG2216-treated group, the vehicle-treated group showed very low level of plasma cell differentiation (Supplemental Figure 3). Furthermore, CpG2216 did not significantly enhance CD19^+^ cell proliferation in PBMCs culture from IgAN patients compared with healthy controls (12.37 ± 1.1% versus 10.44 ± 0.74%, *p* = 0.49). However, we found that CpG2216 stimulation demonstrated superior capability in directing CD19^+^ B cells differentiating into plasma cells in PBMCs culture of IgAN patients, resulting in 50% increase of plasma cell generation than healthy controls (4.56 ± 0.6% versus 3.06 ± 0.37%, *p* = 0.04) (Figure 4(b)). These data suggested that the activation of TLR9 of pDCs from IgAN patients was more potent in promoting the differentiation of plasma cells, as compared with healthy controls.

We also investigated *in vivo* abundance of CD19^+^ cells and plasma cells in IgAN patients. Consistent with ex vivo results, the percentage of CD19^+^ cells was not significantly higher in freshly isolated PBMCs of IgAN patients than in healthy controls (8.16 ± 0.79% versus 10.10 ± 0.99%, *p* = 0.23), but the percentage of plasma cells in freshly isolated PBMCs was about 56% higher in IgAN patients than in healthy controls (0.39 ± 0.04% versus 0.25 ± 0.05%, *p* = 0.04) (Figure 4(c)).

### 3.3. IFN-*α* Treatment Led to More Plasma Cell Differentiation and IgA1 Production from B Cells of Healthy Donors

**Figure 5:**
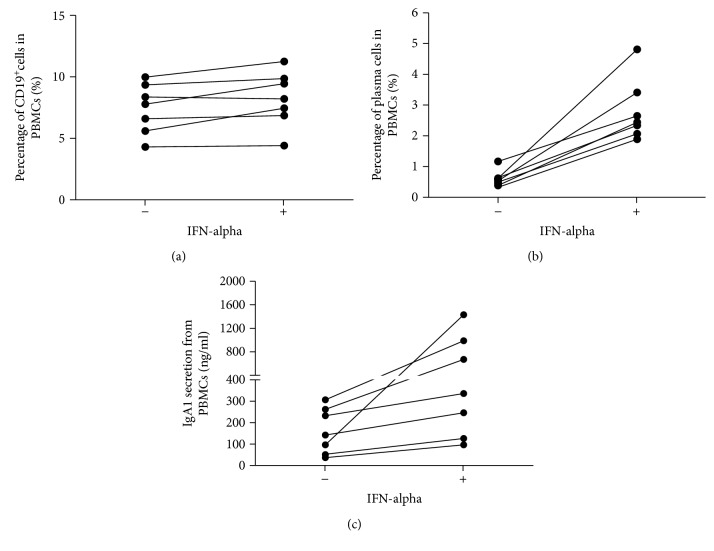
IFN-*α* promote the differentiation of plasma cells from PBMCs ex vivo. PBMCs of different healthy donors (*n* = 7) were prepared by ficoll-paque density centrifugation and cultured with or without IFN-*α* (2000 IU/ml) plus anti-IgM antibody for 6 days, followed by surface markers labeling and analysis in flow cytometry. (a) The percentage of CD19^+^ cells in PBMCs after IFN-*α* treatment for 6 days. (b) The percentage of plasma cells in PBMCs after IFN-*α* treatment for 6 days. (c) IgA1 secretion from PBMCs after IFN-*α* treatment for 6 days.

To test whether IFN-*α* was important for plasma cell differentiation, PBMCs of healthy donors were treated with or without IFN-*α* (2000 IU/ml) for 6 days. As shown in Figure 5, the proliferative effects of IFN-*α* on CD19^+^ cell proliferation were only observed in 3 out of 7 donors (Figure 5(a)), whereas the proliferative effects of IFN-*α* on plasma cell generation were all observed in 7 out of 7 donors (Figure 5(b)). We also found that IgA1 synthesis was increased in all 7 donors (Figure 5(c)). This result indicated that IFN-*α* exerted a positive impact on plasma cell differentiation and IgA1 production, but not on CD19^+^ cell proliferation.

### 3.4. IFN-*α* Promoted Inflammatory Gene Expression in Human Mesangial Cells and Enhanced Transendothelial Migration of Human CD4^+^ Cells and CD14^+^ Cells

**Figure 6:**
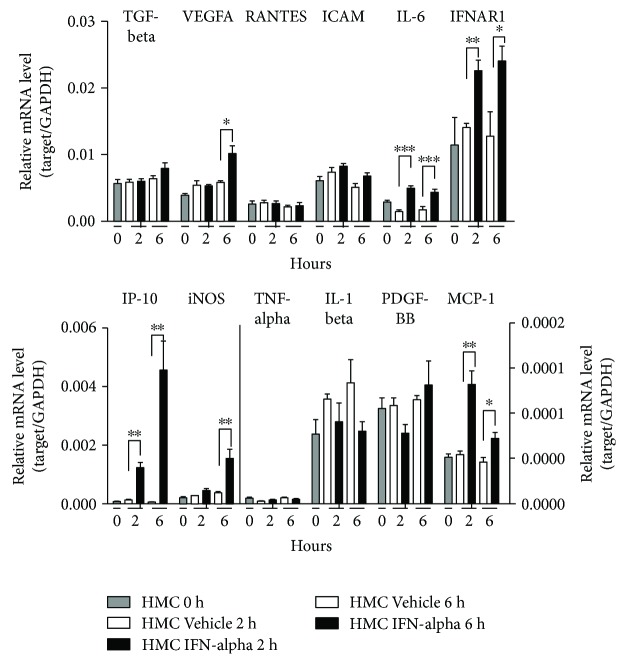
Regulation of gene expression by IFN-*α* in human mesangial cells. Human mesangial cells were subjected to serum starvation for 6 h, followed by IFN-*α* treatment (4000 IU/ml) for varied time points (0 min, 2 h, and 6 h). The mRNA levels of TGF-beta, VEGFA, RANTES, ICAM, IL-6, IFNAR1, IP-10, iNOS, TNF-*α*, IL-1*β*, PDGF-BB, and MCP-1 were measured by real-time PCR. This experiment was repeated independently at least three times, and representative data are shown. ^∗^ *p* < 0.05, ^∗∗^ *p* < 0.01, and ^∗∗∗^ *p* < 0.001.

To test whether IFN-*α* imposes provocative influence on the expression of growth factors, proinflammatory factors, chemokines, and bioactive molecules in human mesangial cells, we analyzed the expression of IFNAR1, TGF-*β*1, VEGFA, PDGF-BB, RANTES, ICAM, IP-10, MCP-1, iNOS, IL-6, IL-1*β*, and TNF-*α* after IFN-*α* treatment. As shown in Figure 6, IFN-*α* receptor was richly expressed in human mesangial cells and significantly upregulated after IFN-*α* treatment. IFN-*α* also enhanced the expression of IL-6, IP-10, and MCP-1 in human mesangial cells after 2 h and 6 h of treatment. IFN-*α* imposed a certain effect on the expression of VEGFA and iNOS whereas less effect on the expression of TGF-*β*1, RANTES, ICAM, TNF-*α*, IL-1*β*, and PDGF-BB. Baselines of mRNA levels of chemokines MCP3, VCAM-1, MIP-1*β*, and CCL17 were very low in HMCs as detected by real-time RT-PCR, no matter with or without IFN-*α* treatment (data not shown).

**Figure 7:**
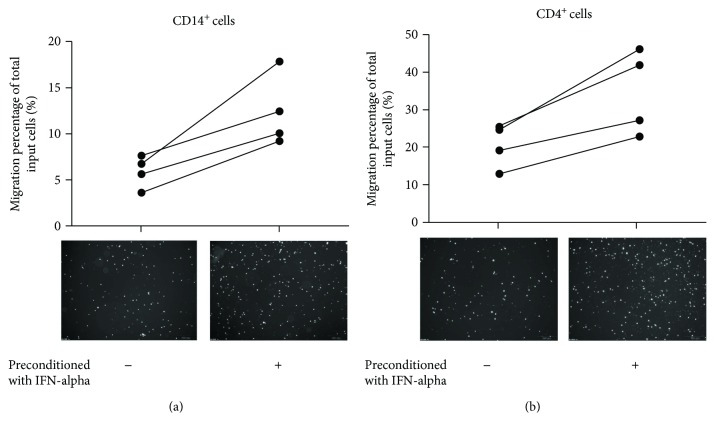
Transendothelial migration of CD14^+^ cells and CD4^+^ cells was promoted by IFN-*α*-conditioned HMC supernatant. HUVEC cells were cultured onto inserts of transwells, and the bottom champers were filled with culture supernatant of HMC preconditioned with recombinant IFN-*α* or vehicles. CD14^+^ cells and CD4^+^ cells were sorted from fresh PBMCs of donors (*n* = 4) by flow cytometry and loaded on the upper wells. After migrating for 6–12 h, migrated cells in the bottom champers were collected and counted in high power field.

The expression levels of chemokines MCP-1 and IP-10 were increased in human mesangial cells after IFN-*α* treatment; therefore, we investigated their ability for attracting leukocytes migrating though endothelial cells. As demonstrated in Figure 7, human CD14^+^ cells and CD4^+^ cells from 4 human donors migrated with higher efficiency in response to conditioned medium of human mesangial cell pretreated with IFN-*α*. For CD4^+^ T cell migration, the numbers of migrated cells of 4 donors attracted by IFN-*α* pretreated supernatant were, respectively, 1.77-, 2.52-, 2.63-, and 1.62-fold of those migrating towards unconditioned supernatant. In the case of CD14^+^ monocytes/macrophages, the numbers of migrated cells attracted by IFN-*α* pretreated supernatant were 1.40-, 1.75-, 1.65-, and 1.87-fold of those migrating towards unconditioned supernatant.

## 4. Discussion

pDC and its secreted cytokine IFN-*α* in autoimmune diseases such as systemic lupus erythematosus and psoriasis have been addressed for their roles in eliciting inflammatory response and tissue damage [48–50]. High levels of IFN-*α* were found in sera of lupus patients, and large numbers of pDCs were found in skins of lupus patients [51–53]. In addition, a global gene expression profiling of PBMCs from lupus patients showed a high number of IFN-*α* responsive genes was abnormally expressed [54]. In the downstream, IFN-*α* leads to the enhanced maturation of dendritic cells, inflamed autoreactive T cell, diminished regulatory T cell activity, and augmented response of B cells [55, 56]. However, the status of pDC and IFN-*α* in the pathogenesis of IgAN has not been well studied. To our knowledge, this is the first study reporting that pDC and its secreted IFN-*α* induced by TLR9 activation enhance plasma cell differentiation and further facilitate monocytes/macrophages and T lymphocyte infiltration across endothelial cell monolayer in IgAN. TLR9 was richly expressed on pDCs, and its activation via CpG-A mainly boosts the synthesis of type I interferon and promotes pDC maturation, while TLR9 activation on B cells via CpG-B induces B cell maturation and Ig synthesis [57, 58]. In a ddY mouse model of IgA nephropathy, treatment with CpG-A but not with CpG-B resulted in increased serum level of IgA-IgG2a immune complex and its glomerular depositions, along with extension of mesangial proliferative lesion [19]. Activation of TLR9 in germinal center B cells induced APRIL synthesis, which induces IgA secretion in IgAN [59]. We found that pDCs were more abundant in IgAN patients and TLR9 activation via CpG-A led to more IFN-*α* production ex vivo, which promote plasma cell differentiation. This result is further supported by the evidence that plasma cells were more abundant in freshly collected peripheral blood cells of IgAN patients. Previous evidence from isolated tonsillar cells showed that TLR9 was expressed in CD19^+^ B cells and BDCA2^+^ pDCs in the tonsils of IgAN patients [20]. Though BDCA2 is a specific marker for pDC in immunostaining, it was reported that BDCA2 was an inhibiting signal for TLR9-mediated IFN-*α* synthesis in pDCs [60] and BDCA2^+^ pDC produced a very low amount of IFN-*α* in lymph node [61]. From our result, it was found that in the tonsil sample of IgAN patients, TLR9 was richly stained in IFN-*α* positive cells, indicating that TLR9 was closely related with IFN-*α* production in IgAN. Combining all these data, it indicates that TLR9 was a critical factor in mediating the aberrant IFN-*α* secretion, B cell differentiation, and Ig synthesis in IgAN. One point worthy to be mentioned is that IFN-*α* was also observed in some tonsillar cells which were negative for TLR9 expression in our study. It is known that TLR7 expressed in pDCs or TLR3 expressed in mDCs or other resident cells were potent mediators for IFN-*α* production [28, 58, 62]. Whether these TLR molecules contributed to augmented IFN-*α* production in IgAN needs further exploration.

As a virus-defending cytokine, IFN-*α* exerts astonishing influences in autoimmune disease systemic lupus erythematosus by activating dendritic cell maturation, antibodies production, immunoglobulin class switching, and T cell subset differentiation [62]. We found that average plasma concentration of IFN-*α* in IgAN patients is about 16 times of that in healthy donors. Besides, ex vivo activation of PBMCs of IgAN patients results in more IFN-*α* synthesis, which directly promotes the differentiation of plasma cells *in vitro.* Supportively, Giordani et al. found that IFN-*α* promoted the plasma cell differentiation from both naive B cells and memory B cells upon TLR9 stimulation [63]. For the underlying mechanism, they found that increased IL-6 expression after IFN-*α* treatment is very important for B cell differentiation [63–65]. Furthermore, IFN-*α* was capable of promoting the expression of CD69, CD86, and CD25 molecules on B cells, as well as inhibiting Fas-mediated apoptosis of B cell, thus leading to low threshold for B cell induction and fast antibody responses [66, 67]. All these data support the notion that IFN-*α* is helpful for expanding B cell survival and plasma cell differentiation in IgAN patients. Our result is beneficial for explaining that bone marrow-resident IgA-producing B cells were upregulated in IgAN patients [68], since these augmented plasma cells may migrate back to bone marrow or inflamed tissue and became long-lived plasma cells which contribute to the autoantibody production in autoimmunity diseases with renal manifestation [69–71]. We also found that IFN-*α* was positively correlated with 24-hour proteinuria in IgAN patients. In murine lupus model, *in vivo* sustained expression of IFN-*α* induced severe proteinuria, immune complex deposition, autoantibody production, and lethal glomerulonephritis [72–74]. Very low dose delivery of exogenous IFN-*α* (<12.5 pg/ml in circulation) could elicit proteinuria in lupus-prone mice [73], which strongly indicated IFN-*α* was very potent in inducing proteinuria. Given all these facts, it is tempting to speculate that higher IFN-*α* level was involved in proteinuria and glomerulonephritis development in IgA nephropathy as it does in lupus.

It is intriguing that IgA1 synthesis in CpG-stimulated PBMCs from IgAN patients was not increased whereas IFN-*α* can promote IgA1 synthesis *in vitro*. An explanation is that besides IFN-*α*, there are other cytokines such as IL-6, IL-12, APRIL, and BAFF elicited by pDC that can influence the synthesis of antibody subsets and plasma cell differentiation [75–79]. However, how these cytokines are influenced by pDC in IgAN patients was unclear and needs further investigation. On the other side, the production of IgG and IgA2 antibodies was enhanced in PBMCs of IgAN patients after CpG2216 stimulation ex vivo. It was reported that pDC combined with CpG induced IgM and IgG synthesis in B cells, and IFN-*α* can further amplify the strength of CpG [63, 80]. Another interesting finding in our study is that renal IgM deposition was positively correlated with both pDC and IFN-*α* abundance in IgAN patients. It was reported that renal IgM deposition was associated with disease progression or glomerular obsolescence in IgAN nephropathy [81, 82]. Besides, we found that renal IgM deposition was positively associated with proteinuria in IgAN patients (data not shown). Although TLR9 activation in pDC was related to IgM synthesis as described above, the underlying mechanism linking the pDC-IFN-*α* axis with renal IgM deposition in IgAN was still unknown.

IgA nephropathy was characterized with renal inflammation accompanied with leukocyte infiltration [83, 84]. T cells and monocytes/macrophages were reported to be predominant infiltrated leukocytes in renal interstitial of IgAN patients, which was significantly correlated with histological injury and renal dysfunction [85–87]. Also, increased MCP-1 expression was found in renal tissues of IgAN patients [88]. Here, we found that IFN-*α* strongly induces mRNA levels of IP-10 (CXCL10) and MCP-1 from mesangial cells *in vitro* and conditioned HMC supernatant with IFN-*α* pretreatment precipitates the transendothelial migration of CD14^+^ and CD4^+^ cells. Considering the fact that IP-10 and MCP-1 were capable of promoting chemotaxis of peripheral blood monocytes and T cells [89–92], enhanced synthesis of IP-10 and MCP-1 from mesangial cells was pivotal in facilitating migration of leukocytes through endothelial cells in IgAN. Other than this, IFN-*α* stimulates the expression of its own receptor IFNAR1, which very possibly generates a positive feedback loop of IFN-*α* signaling. A recent study reported that endothelial cell injury was correlated with proteinuria, hematuria, and renal pathology in a rat IgAN model [93], indicating that integrity of endothelial cell was a key factor for renal function. Combined with our finding, these data support the hypothesis that hyperactivated pDC-IFN-*α* axis exerts strength on promoting the expression of chemokines, mediating leukocyte migration through endothelial cells into renal tissue, and causing proteinuria in IgA nephropathy.

In summary, our results revealed that the pDC-IFN-*α* axis was hyperactivated in patients of IgAN, which further promoted the differentiation of plasma cells and induction of chemokines for assisting migration of T cells and monocytes through endothelial cells. Our data illustrated, at least in part, the mechanism how pDC and IFN-*α* were involved in the pathogenesis of IgAN. As in lupus, high abundance of IFN-*α* and pDC leads to BCR response, B cell proliferation, and antibody production and subsequent proteinuria in lupus [48, 94–96]. It is very possible that IFN-*α* plays a similar role in IgAN patients and lupus patients. Considering the fact that pDC-IFN-*α* blockade therapeutics including anti-IFN-*α* antibody, anti-IFNAR, pDC inhibitors, and TLR9 antagonist are in clinical trials with promising results in lupus [97, 98], controlling the pDC-IFN-*α* axis may also have a beneficial effect for the treatment of IgA nephropathy.

## Figures and Tables

**Table 1:** Clinical and demographic features of IgAN patients and healthy controls.

Feature	pDC		IFN-alpha	
	Healthy controls	IgAN patients	Healthy controls	IgAN patients
Gender (male/female)	12/21	11/21	10/22	9/25
Age (yr)	33.6 ± 9.3	32.5 ± 9.7	35.8 ± 9.6	31.7 ± 9.3
Serum creatinine (*μ*M)		81.3 ± 32.8		69 ± 24
24-hour proteinuria (g/24 h)		1.00 ± 0.78		0.74 ± 0.48
eGFR		98.5 ± 29.1		108.4 ± 23.9

**Table 2:** Association of pDC and IFN-alpha with clinical parameters and renal histopathology in IgAN patients.

Feature	pDC percentage in PBMCs		IFN-alpha protein levels in plasma	
	Pearson's *r*	*p* value	Pearson's *r*	*p* value
Age (yr)	0.05	0.76	0.15	0.39
Serum creatinine (*μ*M)	0.027	0.88	−0.09	0.58
eGFR (ml/min per 1.73 m^2^)	0.005	0.99	−0.02	0.91
24-hour proteinuria (g/24 h)	0.11	0.53	0.34	0.04^∗^
Serum IgA (g/l)	−0.29	0.09	0.07	0.69
Serum IgG (g/l)	0.03	0.85	0.05	0.78
Serum IgM (g/l)	0.22	0.29	0.08	0.67
Serum C3 (g/l)	0.14	0.48	−0.16	0.36
CRP (mg/l)	0.33	0.09	−0.12	0.49
SAA (mg/l)	0.27	0.16	−0.11	0.51
ASO (Ku/l)	0.31	0.11	0.22	0.20
Anti-Dnase B (U/ml)	−0.21	0.28	0.48	0. 005^∗^
Mesangail hypercellularity (M0/M1)	0.09	0.62	−0.07	0.69
Endocapillary hypercellularity (E0/E1)	0.04	0.84	−0.06	0.63
Tubular atrophy/ interstitial fibrosis (T0/T1/T2)	0.03	0.89	0.37	0.03^∗^
Segmental sclerosis (S0/S1)	0.17	0.33	0.11	0.52
Kidney IgA intensity	−0.23	0.20	0.03	0.85
Kidney IgG intensity	0.11	0.55	−0.04	0.81
Kidney IgM intensity	0.45	0.009^∗∗^	0.34	0.04^∗^
Kidney C3 intensity	−0.30	0.09	0.15	0.40

C3: complement 3; CRP: C-reactive protein; SAA: serum amyloid A protein; ASO: antistreptolysin O. ^∗^ *p* < 0.05; ^∗∗^ *p* < 0.001.

## Supplementary Materials

Supplementary 1 Supplemental Figure 1: pDCs respond to CpG2216 stimulation and secrete large amount of IFN-*α* in PBMCs. Freshly isolated PBMCs from donors (*n* = 3) were stained with CD304-APC and CD123-FITC antibodies and subjected to cell sorting. Double positive cells were identified as pDC and the rest cells were identified as PBMCs no pDC (A). Secretion of IFN-*α* proteins in culture supernatant was detected by ELISA after CpG2216 stimulation for 24 hrs (B).

Supplementary 2 Supplemental Figure 2: Synthesis of IFN-*α* induced by different types of oligodeoxynucleotides (ODN) in PBMCs. Freshly isolated PBMCs from donors (*n* = 3) were treated with different ODN as follows. ODN Ctrl: 5′GGGggagcatgctgCGGGGG3′; ODN 2216: 5′GGGggacgatcgtcGGGGGG3′; inhibitory ODN 1: 5′TCCTGGAGGGGTTGT3′; inhibitory ODN 2: 5′ TTTAGGGTTAGGGTTAGGGTTAGG G3′. (Nucleotides in upper letters correspond to phosphorothioate backbone). Culture supernatant was detected for IFN-*α* by ELISA after 24 hrs.

Supplementary 3 Supplemental Figure 3: The plasma cells differentiation in PBMCs was induced by CpG2216. Freshly isolated PBMCs from donors (*n* = 5) were subjected to vehicle or CpG2216 treatment for 6 days, followed by surface makers labeling and analysis in flow cytometry. (A) CD19^+^ cells differentiated into plasma cells (CD19^+^CD38^hi^CD20^lo^) after Vehicle or CpG2216 stimulation for 6 days. (B) Comparison of plasma cell differentiation in Vehicle or CpG 2216-treated PBMCs from donors.
