# Supplementary figures and images for: Increased Abundance of Plasmacytoid Dendritic Cells and Interferon-Alpha Induces Plasma Cell Differentiation in Patients of IgA Nephropathy
Source: Mediators Inflamm. 2017 Dec 18;2017:4532409. doi: 10.1155/2017/4532409 (PMC5748321; doi:10.1155/2017/4532409)

A

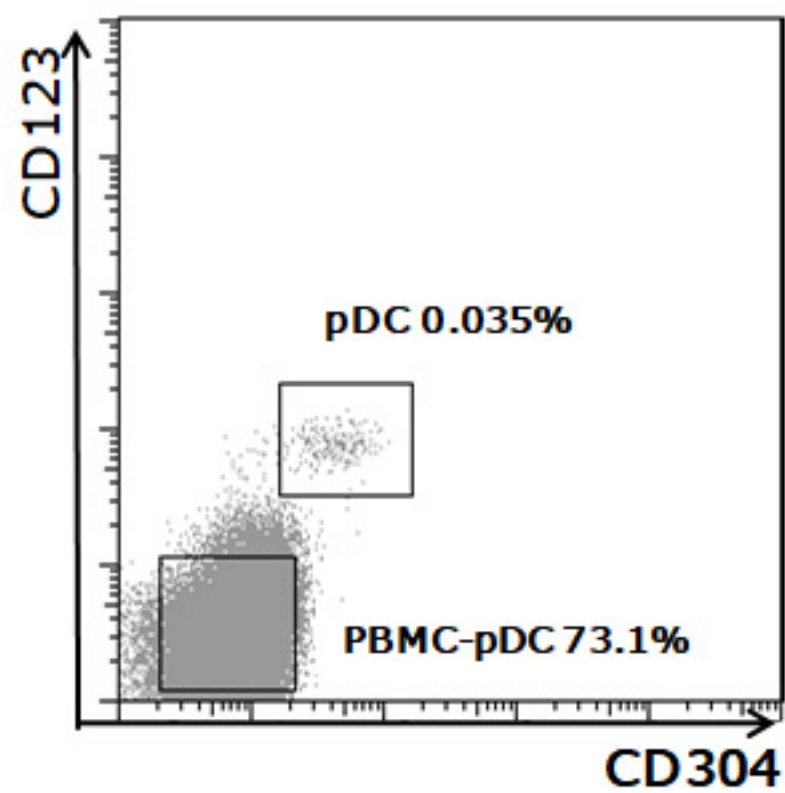

B

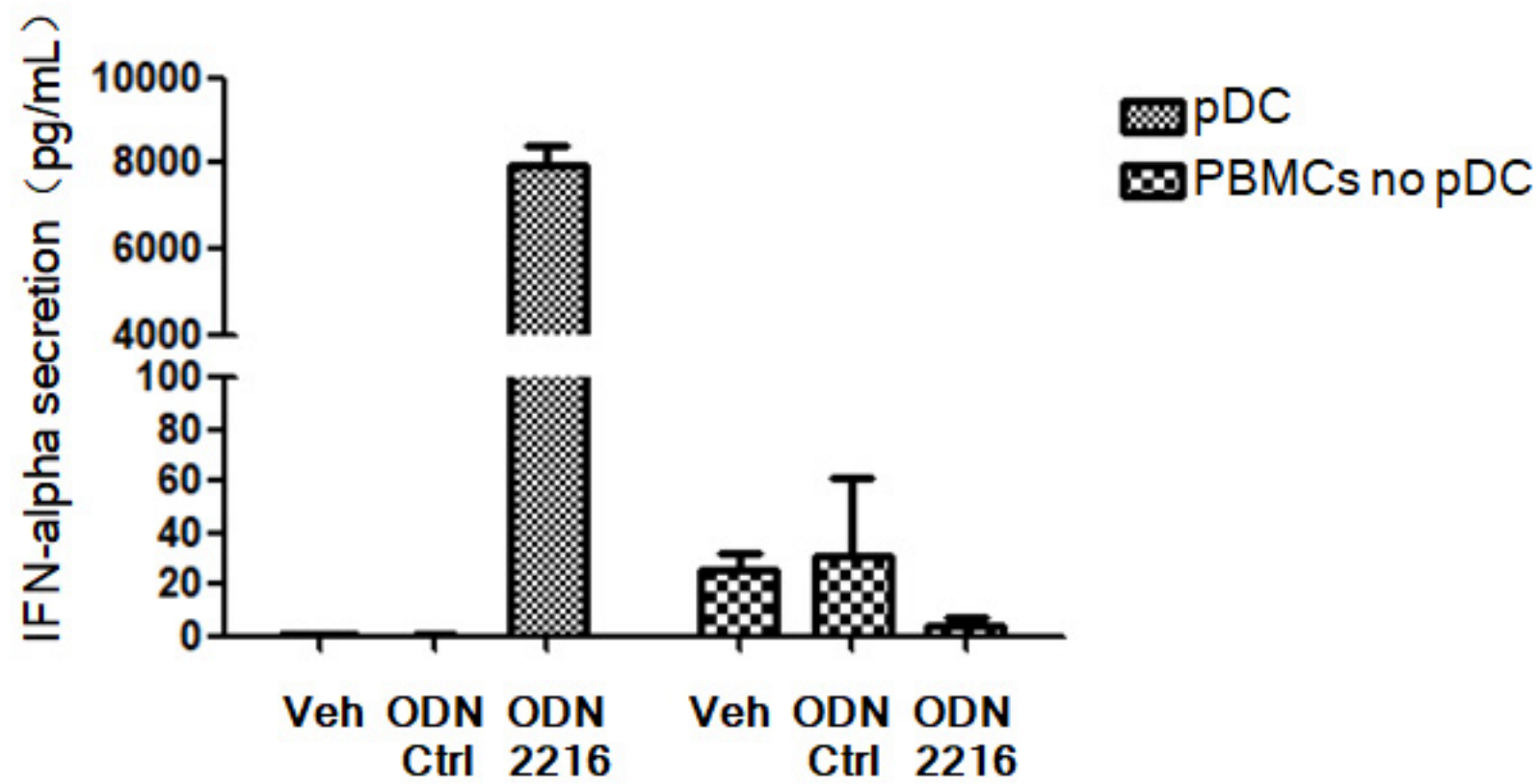

Supplement: Supplementary 1 — Supplemental Figure 1: pDCs respond to CpG2216 stimulation and secrete large amount of IFN-α in PBMCs. Freshly isolated PBMCs from donors (n = 3) were stained with CD304-APC and CD123-FITC antibodies and subjected to cell sorting. Double positive cells were identified as pDC and the rest cells were identified as PBMCs no pDC (A). Secretion of IFN-α proteins in culture supernatant was detected by ELISA after CpG2216 stimulation for 24 hrs (B). [file 4532409.f1.pdf]

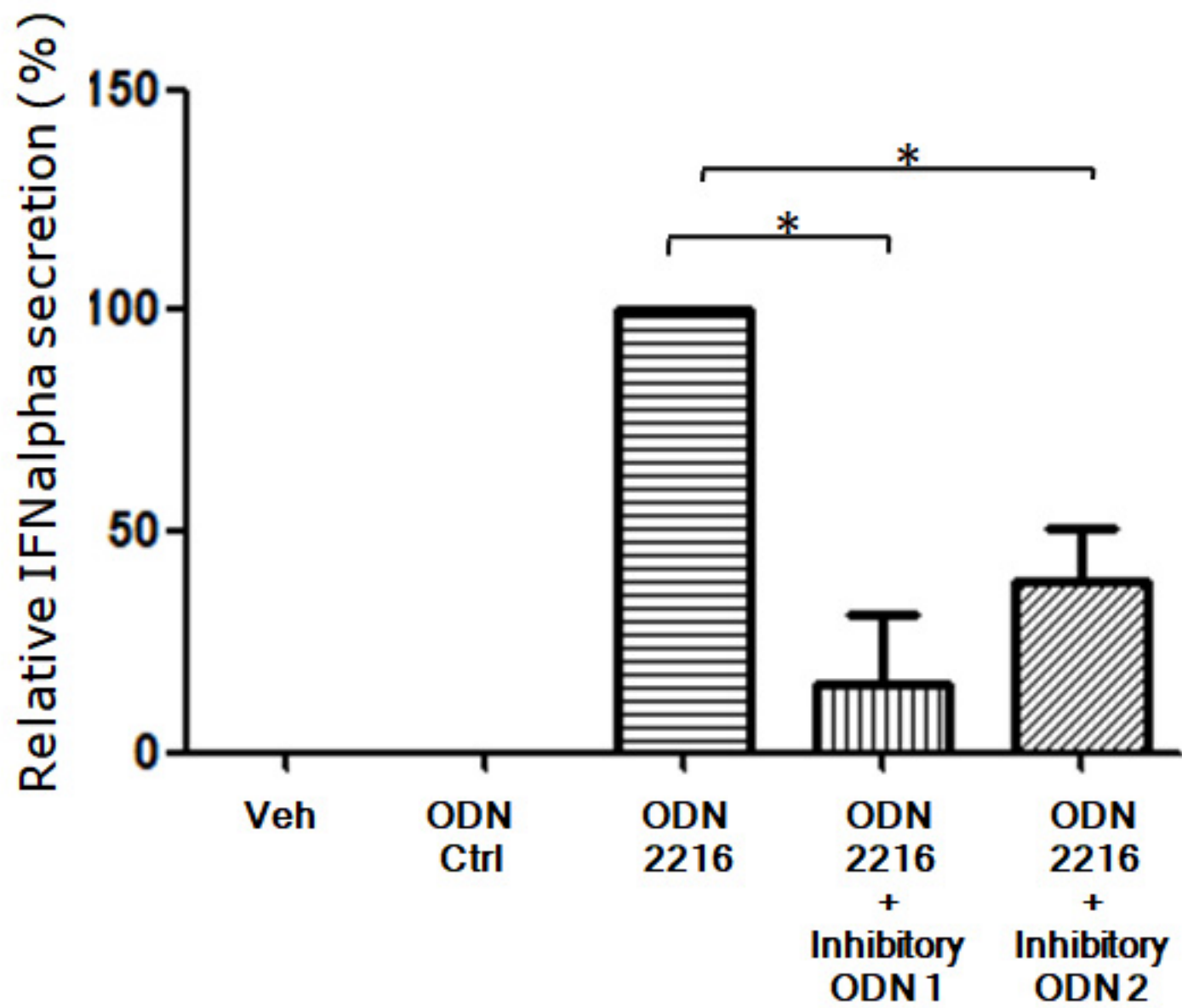

Supplement: Supplementary 2 — Supplemental Figure 2: Synthesis of IFN-α induced by different types of oligodeoxynucleotides (ODN) in PBMCs. Freshly isolated PBMCs from donors (n = 3) were treated with different ODN as follows. ODN Ctrl: 5′GGGggagcatgctgCGGGGG3′; ODN 2216: 5′GGGggacgatcgtcGGGGGG3′; inhibitory ODN 1: 5′TCCTGGAGGGGTTGT3′; inhibitory ODN 2: 5′ TTTAGGGTTAGGGTTAGGGTTAGG G3′. (Nucleotides in upper letters correspond to phosphorothioate backbone). Culture supernatant was detected for IFN-α by ELISA after 24 hrs. [file 4532409.f2.pdf]

**A**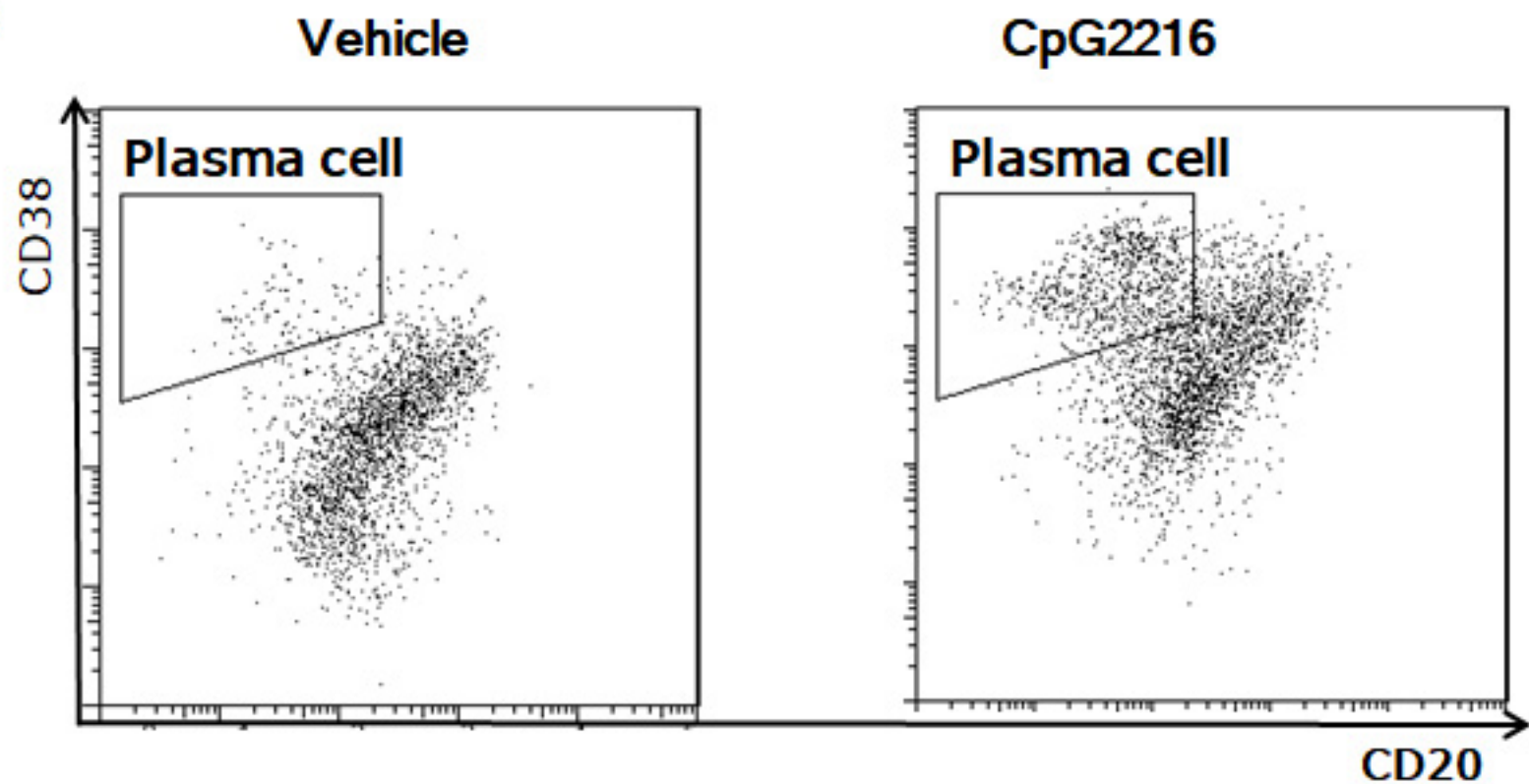**B**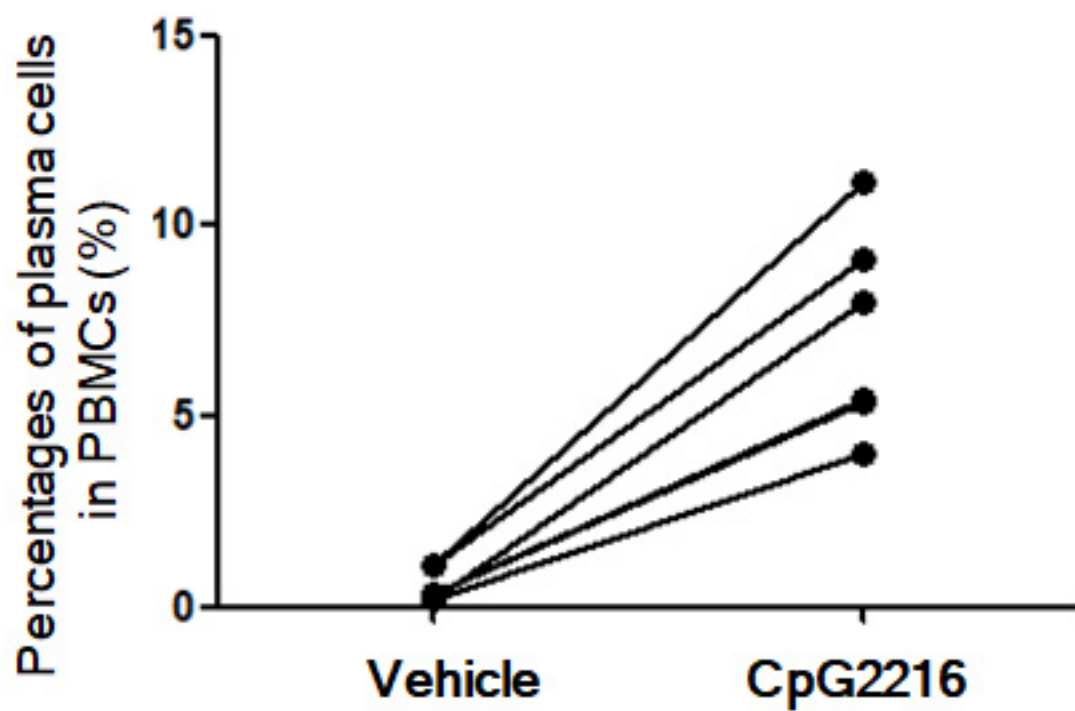

Supplement: Supplementary 3 — Supplemental Figure 3: The plasma cells differentiation in PBMCs was induced by CpG2216. Freshly isolated PBMCs from donors (n = 5) were subjected to vehicle or CpG2216 treatment for 6 days, followed by surface makers labeling and analysis in flow cytometry. (A) CD19+ cells differentiated into plasma cells (CD19+CD38hiCD20lo) after Vehicle or CpG2216 stimulation for 6 days. (B) Comparison of plasma cell differentiation in Vehicle or CpG 2216-treated PBMCs from donors. [file 4532409.f3.pdf]
